# Effect of vitamin A on intestinal mucosal injury in pediatric patients receiving hematopoietic stem cell transplantation and chemotherapy**:** a quasai-randomized trial

**DOI:** 10.1186/s13104-020-05307-8

**Published:** 2020-10-02

**Authors:** Ploy Pattanakitsakul, Nalinee Chongviriyaphan, Samart Pakakasama, Nopporn Apiwattanakul

**Affiliations:** Department of Pediatrics, Faculty of Medicine, Ramathibodi Hospital, Mahidol University, 270 Rama VI Road, Bangkok, 10400 Thailand

**Keywords:** Vitamin A supplementation, Citrulline, Hematopoietic stem cell transplantation, Mucosal injury, Pediatrics

## Abstract

**Objective:**

Vitamin A is involved in maintenance of gut mucosal integrity and normal immune function. However, it is unclear whether these functions of vitamin A have any beneficial effects in patients undergoing hematopoietic stem cell transplantation (HSCT). In this study, we aimed to examine the potential protective effect of vitamin A supplementation on gastrointestinal (GI) mucosal integrity in HSCT recipients using plasma citrulline as a surrogate marker of intestinal integrity.

**Results:**

We performed a quasi-randomized trial in 30 pediatric patients undergoing HSCT. Half (n = 15) of the patients received a single high dose of vitamin A (200,000 IU) before the conditioning regimen was given, and half (n = 15) did not. Clinical data of patients who developed post-transplant complications were recorded for 60 days after HSCT. There were no significant differences in mean plasma citrulline levels on day 7 after HSCT between the treatment and control groups (5.8 vs. 5.9 µmol/L, respectively). The incidence of mucositis and other complications were not different between the two groups within 60 days of HSCT. Vitamin A supplementation prior to HSCT in pediatric patients had no clinical benefit in protecting GI mucosal integrity.

## Introduction

Infection is a major cause of morbidity and mortality during the neutropenic phase in patients undergoing hematopoietic stem cell transplantation (HSCT) [1, 2]. Approximately 40% of stem cell transplant patients develop mucositis following the intensive chemotherapy. In addition to prolonged severe neutropenia, mucosal barrier injury in the gastrointestinal (GI) tract poses additional risks of local bacterial infection and septicemia during the pre-engraftment period [3–9].

Mucositis that occurs following conditioning chemotherapy in these patients is found throughout the GI tract and is generally not directly detectable. Thus, many studies have used plasma citrulline as a noninvasive biomarker of intestinal mucosal damage. Citrulline is an amino acid mainly produced by enterocytes of the small bowel. Decreased levels of plasma citrulline are correlated with significant epithelial small bowel loss [10–12].

Vitamin A plays an essential role in maintaining gut mucosal integrity and immune function [13]. In previous studies of newly diagnosed children with malignancy, vitamin A-deficient patients tended to have more serious febrile episodes, oral mucositis and infections compared with children who had normal vitamin A status [14, 15]. Furthermore, a study in vitamin A-deficient rats revealed that vitamin A supplementation had a protective effect on the intestinal mucosal barrier following intensive chemotherapy [16, 17].

Currently, the World Health Organization (WHO) recommends a single high dose of vitamin A supplementation for severely malnourished children (100,000 IU in 6–11 month-old infants; 200,000 IU in children ≥ 12–71 months of age), especially in Southeast Asia and Africa, where deficiency is a public health problem [18]. Reports have also suggested that vitamin A supplementation can reduce mortality in childhood measles and diarrhea [19]. To date, the potential protective effect of vitamin A supplementation on mucosal injury in HSCT patients remains unclear. We conducted a preliminary study by quasi-randomized trial to examine whether vitamin A could protect against GI mucosal injury after chemotherapy in pediatric patients undergoing HSCT.

## Main text

### Participants

We prospectively enrolled patients aged 1–18 years who underwent HSCT from May 2017 to August 2018 at the Bone Marrow Transplantation Unit, Ramathibodi Hospital, Thailand. Patients were excluded if they had received vitamin A supplementation in the previous 3 months before enrollment or had renal insufficiency (GFR < 50 mL/min), which causes false elevation of citrulline levels [10].

HSCT was performed on the basis of underlying diseases according to our institutional protocols. All participants received conditioning chemotherapy, supportive care and antimicrobial prophylaxis after receiving stem cells.

This trial was approved by the Institutional Ethics Committee, Faculty of Medicine, Ramathibodi Hospital, Mahidol University (ID 03-60-18). All patients and/or their parents provided written informed consent. The study was registered at the Thai Clinical Trials Registry (TCTR20191207002) and partly adhered to CONSORT guidelines.

### Study design

This study was a preliminary, single center, prospective, quasi-randomized, clinical trial. Eligible patients were assigned to one of two groups by 1:1 alternation: 15 patients received a single oral dose of 200,000 IU of vitamin A before the conditioning chemotherpay; the other 15 patients did not receive vitamin A supplementation (Additional file 1: Figure S1). Baseline characteristics including age, sex, body weight, height, diagnosis, donor type and conditioning chemotherapy regimens were collected.

Blood samples were collected before administration of vitamin A, on stem cell infusion day (day 0), and 7 days after HSCT. Plasma was separated by centrifugation and stored at − 80 °C until analysis. Plasma retinol and citrulline levels were analyzed using high-performance liquid chromatography (HPLC) and cation-exchange HPLC techniques, respectively.

All of the patients were followed for a period of 60 days after HSCT. Clinical data including number and consistency of stools, fever and oral mucositis were recorded thrice daily (every 8 h).

### Outcomes

The primary outcome of this study was GI mucosal damage after HSCT indicated by plasma citrulline level. Secondary outcomes, assessed up to 60 days after HSCT, were post-HSCT complications including incidence of febrile neutropenia, oral mucositis, infections, diarrhea, graft-versus-host disease (GVHD), hemorrhagic cystitis, time of neutrophil engraftment, length of hospital stay, and mortality. Incidence of vitamin A toxicity (headache, vomiting, seizure, change in mental status, visual disturbance) was also recorded.

### Definitions

Diarrhea was defined as passage of ≥ 3 watery stools in a 24-h period. Bacterial infection was defined as presence of pathogenic bacteria in cultures of blood, cerebrospinal fluid or > 10^5^ CFU/mL in cultures of urine. Pneumonia was diagnosed by imaging study. Febrile neutropenia was defined as a single oral temperature measurement > 38.3 °C, or a temperature > 38 °C sustained over a 1-h period with absolute neutrophil count < 500 cells/µL or expected to decrease to < 500 cells/µL during the next 48 h. Neutrophil engraftment was defined as an absolute neutrophil count > 500 cells/µL for 3 consecutive days after HSCT. Vitamin A deficiency and insufficiency were defined as plasma vitamin A levels < 20 and 30 µg/dL, respectively.

### Statistical analysis

Between-group comparisons were analyzed using the Mann Whitney U-test or the Student’s *t*-test for continuous data, and the χ^2^ test or Fisher’s exact test for categorical data. Comparisons of plasma vitamin A and citrulline levels between the two groups were performed by mixed linear regression. Statistical significance was defined as a *p* value < 0.05. Statistical analysis of data was performed using the Stata version 15.1 (StataCorp LLC, College Station, TX, USA).

### Results

**Fig. 1:**
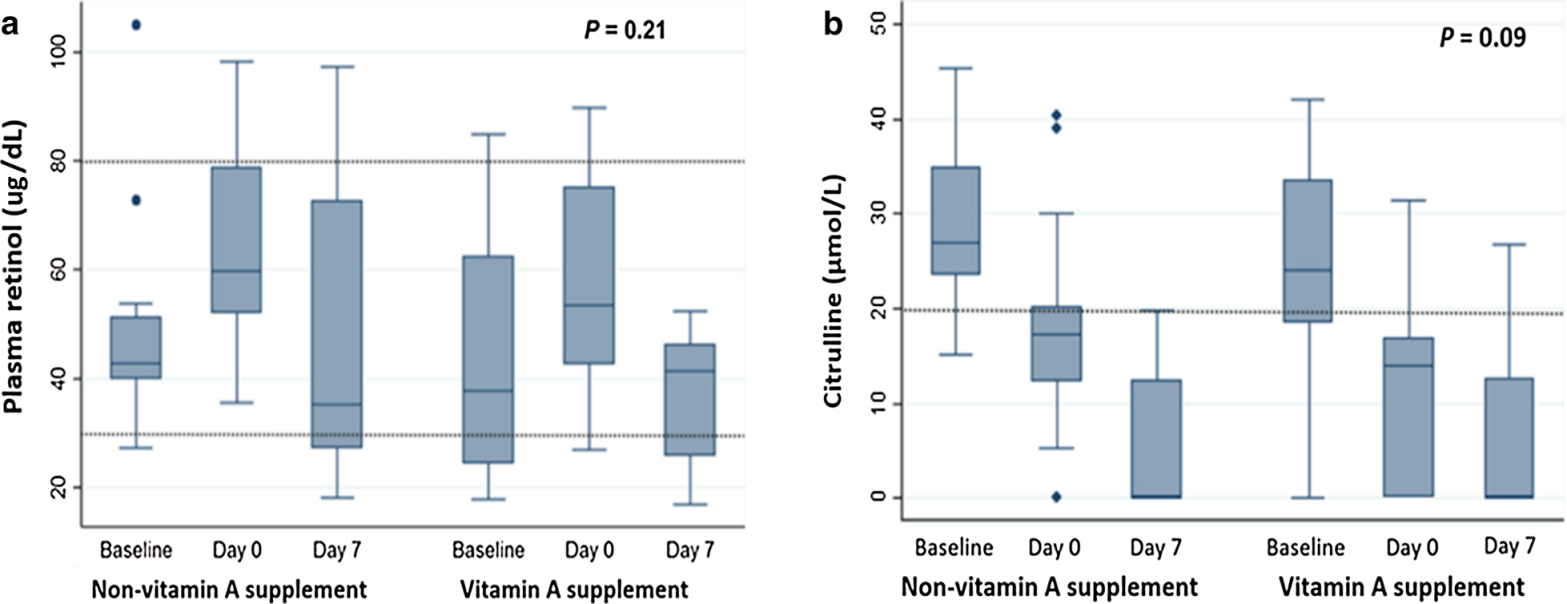
Levels of plasma retinol (**a**) and citrulline (**b**) at baseline, and at days 0 and 7 following hematopoietic stem cell transplantation in the two study groups. The values represent median with interquartile range

The baseline characteristics of the eligible patients are shown in Table 1. The mean age was 9.6 years in the vitamin A group and 7.8 years in the control group. Forty percent of the patients had underlying diseases of malignancy. Two-thirds of the patients underwent haploidentical HSCT. The baseline plasma vitamin A level was similar between the vitamin A (44.1 ± 21.4 µg/dL) and control (47.9 ± 19.3 µg/dL) groups. Five patients in the vitamin A group and two patients in the control group had a vitamin A level < 30 µg/dL (Table 2). No patients had clinical signs of vitamin A deficiency at the time of enrollment. Figure 1a shows that the plasma vitamin A level increased from baseline, peaked at day 0 and decreased at day 7; however, these changes were not different between the two groups.

**Table 1:** Baseline characteristics of the two groups of study patients

Characteristics	Study groups	
	Vitamin A supplementation (n = 15)	Non-vitamin A supplementation (n = 15)
Age, mean years ± SD	9.6 ± 4.3	7.8 ± 2.5
Male, n (%)	10 (66.7)	7 (46.7)
Underlying disease, n (%)		
Malignant disease	7 (46.7)	5 (33.3)
Nonmalignant disease	8 (53.3)	10 (66.7)
Type of HSCT, n (%)		
Haploidentical	10 (66.7)	10 (66.7)
Non-haploidentical	5 (33.3)	5 (33.3)
Weight for height, mean % ± SD	102.4 ± 18.63	97.9 ± 26.07
Baseline plasma retinol (µg/dL), mean ± SD	44.1 ± 21.4	47.9 ± 19.3
Plasma retinol < 30 µg/dL, n (%)	5 (33.3)	2 (13.3)
Baseline plasma citrulline (μmol/L), mean ± SD	25.7 ± 10.8	29.2 ± 8

*SD* standard deviation, *HSCT* hematopoietic stem cell transplantation

**Table 2:** Incidence and duration of secondary outcomes within 60 days of hematopoietic stem cell transplantation in the two groups of study patients

Outcomes	Study groups		*P* value
	Vitamin A supplementation (n = 15)	Non-vitamin A supplementation (n = 15)	
Febrile neutropenia, n (%)	11 (73.3)	11 (73.3)	1
Oral mucositis, n (%)	14 (93.3)	15 (100)	1
Oral mucositis severity grade 3–4, n (%)	6 (40)	9 (60)	0.27
Diarrhea, n (%)	14 (93.3)	14 (93.3)	1
Hemorrhagic cystitis, n (%)	5 (33.3)	5 (33.3)	1
Graft-versus-host disease, n (%)	6 (40)	7 (46.7)	1
Bacterial infection, n (%)	1 (6.7)	2 (13.3)	1
Viral infection, n (%)	10 (66.7)	6 (40)	0.14
Day of neutrophil engraftment, mean days ± SD	14 ± 2.8	14.2 ± 3.9	0.87
Duration of febrile neutropenia^a^, days	5 (2–10)	6.5 (1–21)	0.8
Duration of mucositis, mean days ± SD	9 ± 3.8	9 ± 4.7	0.96
Duration of diarrhea^a^, days	11.5 (2–44)	15.5 (2–37)	0.42
Duration of need of TPN, days ± SD	13 ± 7.1	11 ± 6.6	0.42
Duration of hospitalization after day 0^a^, days	29 (18–83)	24 (15–54)	0.13
Mortality, n (%)	0	2 (13.3)	0.48

^a^Median (min–max)

*SD* standard deviation, *TPN* total parenteral nutrition

### Effect of vitamin A supplementation on plasma citrulline levels

Low plasma citrulline (< 20 μmol/L) indicates intestinal mucosal injury [12]. In this study, plasma citrulline decreased following HSCT, with mean plasma citrulline levels reaching their lowest points at day 7 in both groups. There was no statistically significant difference in the change in citrulline levels between the two groups (*p* = 0.09; Fig. 1b).

### Complications after HSCT

There was no significant difference in the incidence of febrile neutropenia, oral mucositis, invasive bacterial infections, diarrhea, hemorrhagic cystitis, graft-versus-host disease or death between the two groups (Table 2). The severity of oral mucositis graded by the WHO scale [5] was also not different between the two groups (Table 2).

Two patients died during the study period, both belonging to the control group. The first patient was a 5-year-old girl with underlying neuroblastoma who underwent haploidentical HSCT and developed toxic leukoencephalopathy 18 days later. The second patient was an 8-year-old girl with underlying acute myeloblastic leukemia who also underwent haploidentical HSCT and developed massive GI bleeding 48 days after HSCT. The cause of bleeding could not be determined.

The mean durations of febrile neutropenia and diarrhea tended to be shorter in the vitamin A group, but these differences were not statistically significant (Table 2).

### Incidence of vitamin A toxicity

None of the enrolled patients had clinical features of vitamin A toxicity. Vitamin A supplementation in patients with normal vitamin A status at baseline did not lead to any adverse events. Moreover, plasma vitamin A at day 7 after HSCT did not reach a level considered toxic (> 100 µg/dL) [20] in either group (Fig. 1a).

### Discussion

This preliminary trial on vitamin A supplementation in pediatric patients undergoing HSCT did not show a beneficial effect of vitamin A on intestinal mucosal integrity. We found a prevalence of vitamin A insufficiency of 23% in our study population. Conditioning chemotherapy can markedly impair intestinal mucosal integrity, as indicated by a reduction in plasma citrulline level. While vitamin A supplementation in this study did not rescue this effect, it did show a tendency of beneficial effects in terms of reduced duration of diarrhea and febrile neutropenia.

There are several possible explanations for why supplementation of vitamin A, proven to be an essential nutrient for the maintenance of gut mucosal integrity and immune function [13, 21], did not show a significant beneficial effect on mucosal integrity in our pediatric patients undergoing HSCT. We found that the prevalence of vitamin A deficiency in this study was lower than a previous study [15]. Therefore, the efficacy of a single high dose of vitamin A might not have been observable. Another possibility is that the dose of 200,000 IU vitamin A in this study was not sufficient to rescue the mucosal integrity, as evidenced by the patients in both groups showing similar decreases in plasma citrulline levels following HSCT. The fact that no patients who received vitamin A supplementation had a toxic level of vitamin A after supplementation implied that the dose could be safely increased. Hussey and Klein [22] showed that high-dose vitamin A supplementation (400,000 IU) led to markedly diminished mortality and morbidity in in children hospitalized with severe measles in South Africa. However, 92% of the children in their study had hyporetinemia (serum vitamin A level < 20 µg/dL).

Since one animal study showed that exogenous retinoic acid significantly increased expression of the gut-homing molecules, CCR9 and α4β7, and accumulation of CD4 + and CD8 + expression in the gut mucosa, which could potentially increase the risk of gut GVHD in patients who receive exogenous vitamin A [23]. However, the most recent study conducted by Gjærde et al. [24] showed that pretransplant vitamin A level was not associated with incidence of acute GVHD or gastrointestinal GVHD. Our result supported this study that vitamin A supplementation did not affect the incidence of acute GVHD in patients following HSCT. The discrepancy between the animal study and the studies in human may be due to the fact that all-trans-retinoic acid was injected directly into peritoneum in the animal study. This may not represent what actually occurs in human body.

Of note, we observed that plasma vitamin A levels tended to increase at day 0 in patients in both groups compared with baseline levels. This result might reflect an improved nutritional status of the patients after being hospitalized. The patients’ diets were well controlled by hospital dieticians, and may have elevated their vitamin A levels. This might have played a role in masking the potentially beneficial effects of vitamin A supplementation on our outcomes of interest. However, plasma vitamin A levels decreased at day 7 after HSCT. This may have been related to low nutritional intake and decreased intestinal absorption due to presence of mucositis or higher vitamin A utilization [15]. Although plasma citrulline levels were lowest at day 7 after HSCT, reflective of ongoing intestinal mucosal damage, plasma vitamin A levels were still in the normal range. We speculate that the intestinal mucosal integrity in our patients was also influenced by other factors, including zinc level [25], inflammation, infection [26], gut microbiota [27, 28] and host immune response [13]. Another possibility is that a higher plasma concentration of vitamin A was needed to rescue the intestinal damage.

Given that the serum vitamin A and citrulline levels decreased from day 0 to day 7, it is worth exploring further whether vitamin A supplementation around this time period could improve intestinal integrity. It would also be interesting to investigate whether plasma vitamin A levels continue to decrease after day 7 post-HSCT. If so, supplementation of vitamin A at this time point may show benefits in improved outcomes of HSCT.

### Conclusion

This preliminary study showed that vitamin A supplementation in the preconditioning period had no clinical benefit on GI mucosal integrity in pediatric patients undergoing HSCT. Several factors could be involved in modulating the effects of vitamin A on GI mucosal integrity, including the status of other vitamins and minerals and the gut microbiota. The dose and appropriate timing for administration of vitamin A supplementation should be investigated further.

## Limitations

- The results of this study are preliminary. A larger sample size is needed to examine the potential benefits of vitamin A in the reduction of duration of febrile neutropenia and diarrhea in this patient population.
- This study was not a true randomized clinical trial. However, preliminary results from this study could be a basis for a randomized clinical trial with appropriate sample size.
- A dose–response effect of vitamin A was not performed.

## Supplementary information

**Additional file 1: Figure S1** Flowchart of enrollment of pediatric study patients receiving hematopoietic stem cell transplantation and chemotherapy.

**Additional file 2:** Raw data of the results in the study.

## Data Availability

All data generated and analyzed during this study are included in this published article (in Additional files: 1, 2).

## Abbreviations

WHO: World Health Organization
GI: Gastrointestinal
HSCT: Hematopoietic stem cell transplantation
TPN: Total parenteral nutrition
