# Supplementary figures and images for: Effect of vitamin A on intestinal mucosal injury in pediatric patients receiving hematopoietic stem cell transplantation and chemotherapy: a quasai-randomized trial
Source: BMC Res Notes. 2020 Oct 2;13:464. doi: 10.1186/s13104-020-05307-8 (PMC7532573; doi:10.1186/s13104-020-05307-8)

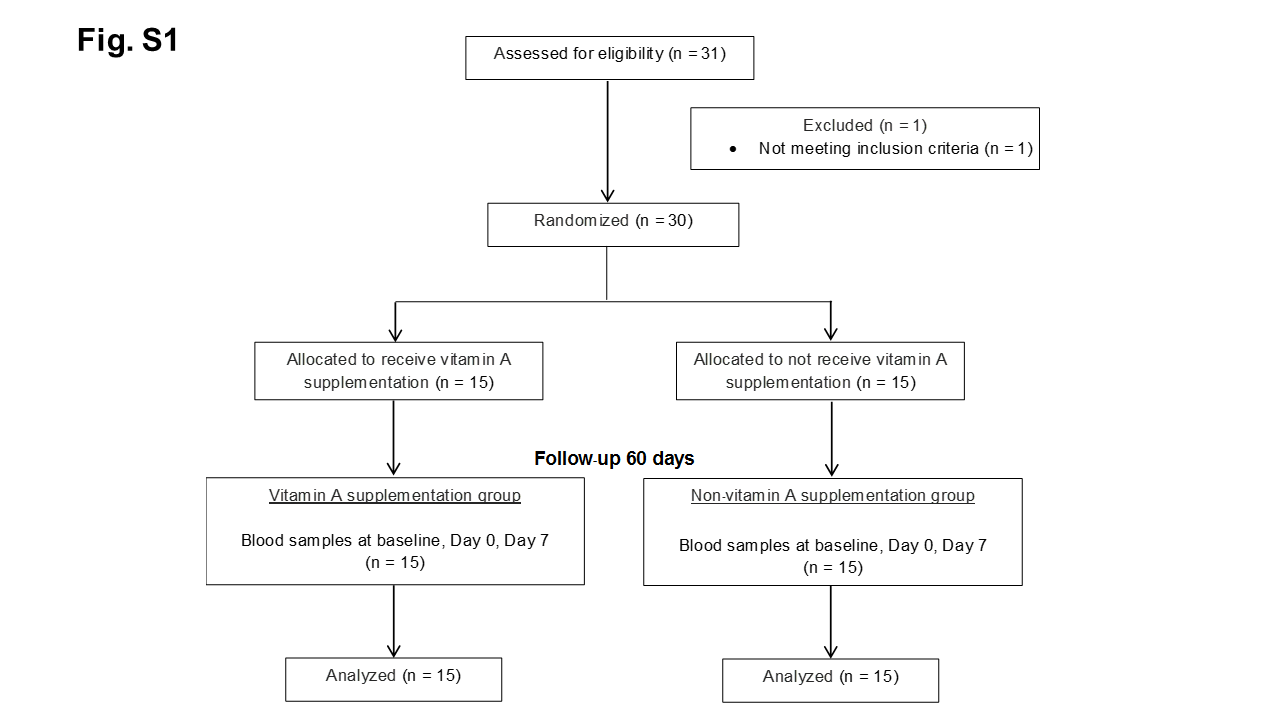

Supplement: Supplementary file 1 — Additional file 1: Figure S1 Flowchart of enrollment of pediatric study patients receiving hematopoietic stem cell transplantation and chemotherapy. [file 13104_2020_5307_MOESM1_ESM.tif]
